# Emerging from COVID-19: Lessons for Action on Climate Change and Health in Cities

**DOI:** 10.1007/s11524-020-00501-2

**Published:** 2021-03-01

**Authors:** James Milner, Mike Davies, Andy Haines, Rachel Huxley, Susan Michie, Lawrie Robertson, José Siri, Paul Wilkinson

**Affiliations:** 1 Centre on Climate Change and Planetary Health, London School of Hygiene and Tropical Medicine, Keppel Street, London, WC1E 7HT UK; 2 Department of Public Health, Environments and Society, London School of Hygiene and Tropical Medicine, 15-17 Tavistock Place, London, WC1H 9SH UK; 3 Institute for Environmental Design and Engineering, University College London, Central House, 14 Upper Woburn Place, London, WC1H 0NN UK; 4 Department of Population Health, London School of Hygiene and Tropical Medicine, Keppel Street, London, WC1E 7HT UK; 5 C40 Cities Leadership Group, 3 Queen Victoria Street, London, EC4N 4TQ UK; 6 Centre for Behaviour Change, University College London, 1-19 Torrington Place, London, WC1E 7HB UK; 7 Buro Happold, 17 Newman Street, London, W1T 1PD UK; 8 Our Planet Our Health, Wellcome Trust, 215 Euston Road, London, NW1 2BE UK

## Introduction

The COVID-19 pandemic has required health protection responses with far-reaching consequences for society, livelihoods, and the wider economy. Future enquiries will in time evaluate the success of responses at all scales. But emerging lessons highlight immediate implications for addressing the growing climate crisis through a recovery from COVID-19 that advances population health, economic regeneration and climate action [1].

Cities are where many of the most critical actions for health, greenhouse gas (GHG) emissions reduction, resilience and risk reduction must be taken, supported by national governments, multi-lateral agencies and other stakeholders [2]. Rapid decarbonisation across all sectors of society is needed over this decade—further delay will seriously reduce the possibility of achieving the targets set out in the Paris Agreement [3]. Now is therefore an especially important juncture for cities to act for both the near-term imperatives of the post-COVID recovery and the long-term welfare of their residents and the planet.

## What COVID-19 Has Shown

COVID-19 has illustrated how quickly and dramatically the normal functioning of society can be changed by disruptive forces. Briefly, it has shown that:

- All societies, even the most advanced, are inherently vulnerable to unexpected (or inadequately planned for) disruptions, which can have severe and wide-ranging domino effects, especially when necessary infrastructure and preparedness are absent [4].
- Scientific evidence is crucial in guiding policy and needs to be managed and communicated in a way that conveys uncertainty, yet inspires broad acceptance by the population [5].
- The negative effects of COVID-19 are unequally distributed within societies, with appreciably greater impacts in socio-economically deprived and vulnerable populations [6].
- The social and economic disruptions of COVID-19 have yielded temporary improvements in air quality in some locations because of reduced fossil fuel use [7] and inspired calls for accelerated action for a zero-carbon economy [8, 9].
- Large-scale action to change public behaviours is possible at breath-taking pace in an emergency, but it has major adverse socioeconomic impacts that could be reduced or avoided through proactive planning.

## Actions to Support Health and Sustainability

A key imperative for recovery is to target investments at actions that support long-term health and sustainability, as well as resilience. Below we highlight seven areas that deserve attention because they present opportunities to build a positive legacy for people and the planet.

1. Embedding action to promote active travel and decarbonise transport

Concern over COVID-19 means that use of public transport will be diminished for the immediate future [10]. A shift to increasing reliance on private motor vehicles would however increase congestion, inequities, pollution and GHG emissions. In contrast, increased walking and cycling, where feasible, is not only an essential part of transport solutions but is widely recognized as strategically desirable for the long-term from both environmental and health perspectives [11]. The increased readiness to take up active travel during the COVID-19 pandemic presents a rare opportunity to maximize the implementation of initiatives to promote walking and cycling—including temporary widening of pavements or closures of roads—and make them permanent [12]. Cities around the world have implemented measures to support pedal power over fossil power [12]. These measures will be yet more valuable if also used to catalyse further action. Improvements in public transport infrastructure and scheduling (coupled with altered work and study patterns) will be vital to encourage a return to safe use of public transport, with physical distancing where possible. Most fundamentally, creative thinking is needed to allow people to meet their needs close to their places of residence—an aspiration encapsulated by the concept of the ‘15-min city’ that envisages employment and essential services being available within a short journey [13, 14].

2. Different modes of working

Many people, primarily in higher income settings, have through necessity substituted electronic means of communication for travel during the crisis. For many reasons (including diminished social interactions, mental health and physical activity), it is not desirable that people are forced to adopt socially isolated lifestyles [15]. But there have been clear and widely accepted benefits: some have welcomed the shift to more home-based working and would prefer to rebalance their commitments towards more home working in future [16]. It is clear that much work-related travel (long-distance travel in particular) is not always essential and avoiding it may in some circumstances aid productivity while helping to reduce environmental impacts. It is therefore appropriate to consider how work and travel behaviour may be rebalanced by technology and policy innovation. This should entail consideration of the differential impacts of work patterns on men and women and on those with caring responsibilities [17]. Actions to facilitate working in local communities—for example, local working hubs where employers hire space and time—could reduce travel and help to improve local environments and social cohesion.

3. Using health to motivate accelerated progress to a zero-carbon economy

One consequence of COVID-19 restrictions has been tangible improvements in air quality in some locations when large sections of the economy were temporarily suspended [7], as demonstrated, for example, via satellite imagery [18]. While such changes have largely reversed as economic activity recovers, recognition of positive developments that have emerged during the COVID-19 pandemic (not only less pollution but, e.g. greater contact with nature, social cohesiveness and volunteerism) and their associated health benefits provides an opportunity to win support for the acceleration of low-carbon actions [19]. Air pollution exposure is a risk factor for many conditions—such as heart disease, stroke and diabetes—that increase the risk of death from COVID-19 [20]. It would also be opportune to explore whether some changes necessitated by COVID-19 may be worth retaining. For example, the National Health Service in England has adopted a ‘digital first’ principle for many primary care and outpatient consultations [21]. For some conditions, virtual consultations may be appropriate in the long-term and could provide efficiency savings, including reduced GHG emissions [22].

4. Improvement in housing quality, especially for low income settings

The confinement of large populations to home for prolonged periods has underscored the importance of the home as a place of safety and potential health promotion. Overcrowding, limited space and inadequate facilities for hygiene and maintenance of a healthy indoor and outdoor environment prevent this potential from being realized [23]. Such risks are likely to be especially high in the overcrowded and insanitary conditions of congested informal settlements that are common in many cities around the world [24]. Implementing clean cooking solutions and improved handwashing will be the vital parts of COVID-19 responses in these settings [25]. Beyond the spread of infection, the interaction of COVID-19 with multiple other environmental, sanitation and safety risks makes such environments especially hazardous [26]. The health impacts of heat are likely to rise in future where populations are confined to home during hot summers, even where housing is of better standard [27]. These challenges should stimulate the strengthening of policies to improve housing quality for health and environmental sustainability, especially for poorer populations. In many settings, there remains a great need and a major opportunity for building energy efficiency retrofits, especially for social and public sector housing that could create large numbers of jobs as well as deliver healthier, lower carbon houses.

5. Improving the resilience of food and other critical supply infrastructures

Restrictions on movement to combat COVID-19 have highlighted the importance and vulnerability of the logistical systems and workforces serving our cities. The recognition of food system workers as ‘key workers’ marks a step forward. In the longer term, cities will need to monitor and, where appropriate, intervene to reduce the vulnerability of food supplies to combined simultaneous global and local disruptions [28]. An important part of this response may be to encourage diversification of sources, as well as reducing food waste and increasing exploitation of local, seasonal production [29], while encouraging a shift towards healthy sustainable diets to limit environmental impacts [30].

6. Improving local environments for health

Another consequence of COVID-19 restrictions has been to confine people to their local areas for outdoor activities, including recreation. There is a wealth of evidence on how green infrastructure and urban design can help promote health and well-being [31]. Green infrastructure also has an important role in responses to climate change, for example, by providing shade and reducing heat-related deaths, assisting in flood protection, reducing demand for refrigerated air conditioning and promoting active travel [32–34]. However, access is often limited and unequally distributed [35]. The experience of COVID-19 should therefore give further impetus to developing local green space that is safe and accessible to all.

7. Strengthening of public health services

COVID-19 has been an extraordinary challenge for public health systems globally, which rarely have the capacity to respond at the required pace and scale, despite long-standing recognition of the threat of global pandemics [4]. Strengthening those systems is vital in the face of the certainty of future threats and must take account of our global interdependence, recognizing that problems for any country in responding to the epidemic threat represent a challenge to all countries. This interconnectedness underlines the need for support from well-prepared and resourced countries to the least resourced. Moreover, the issue is not only confined to pandemics. Disruptive regional risks and cascading, interdependent impacts are also likely with climate change, which will require preparedness to ensure risks to health and the economy are minimized [36]. Given the context of the recent dislocations, it is critical that health systems demonstrate leadership on climate change, not only in terms of emergency responses but in long-term planning for adaptation, and by taking steps to reduce the often substantial environmental footprint of their own operations.

## Author Roles

This commentary was written by an interdisciplinary group of experts that draw on their international experience in research on urban health and sustainability. They are collaborators in a research consortium entitled ‘Complex Urban Systems for Sustainability and Health’ (CUSSH), which seeks to understand how urban transformation can be accelerated to meet imperatives for planetary and population health. The first draft of the paper was written by PW and edited by JM. All other authors contributed to editing.
